# Reading m^6^A marks in mRNA: A potent mechanism of gene regulation in plants

**DOI:** 10.1111/jipb.13781

**Published:** 2024-10-04

**Authors:** Thi Kim Hang Nguyen, Hunseung Kang

**Affiliations:** ^1^ Department of Applied Biology, College of Agriculture and Life Sciences Chonnam National University Gwangju 61186 Korea

**Keywords:** epitranscriptomics, m^6^A modification, m^6^A reader, RNA metabolism, YTH

## Abstract

Modifications to RNA have recently been recognized as a pivotal regulator of gene expression in living organisms. More than 170 chemical modifications have been identified in RNAs, with *N* ^6^‐methyladenosine (m^6^A) being the most abundant modification in eukaryotic mRNAs. The addition and removal of m^6^A marks are catalyzed by methyltransferases (referred to as “writers”) and demethylases (referred to as “erasers”), respectively. In addition, the m^6^A marks in mRNAs are recognized and interpreted by m^6^A‐binding proteins (referred to as “readers”), which regulate the fate of mRNAs, including stability, splicing, transport, and translation. Therefore, exploring the mechanism underlying the m^6^A reader‐mediated modulation of RNA metabolism is essential for a much deeper understanding of the epigenetic role of RNA modification in plants. Recent discoveries have improved our understanding of the functions of m^6^A readers in plant growth and development, stress response, and disease resistance. This review highlights the latest developments in m^6^A reader research, emphasizing the diverse RNA‐binding domains crucial for m^6^A reader function and the biological and cellular roles of m^6^A readers in the plant response to developmental and environmental signals. Moreover, we propose and discuss the potential future research directions and challenges in identifying novel m^6^A readers and elucidating the cellular and mechanistic role of m^6^A readers in plants.

## Graphical abstract

This review examines N^6^‐methyladenosine (m^6^ A) metabolism, including how m^6^A readers, writers, and erasers recognize and interpret m^6^A modification on mRNAs; how they regulate the stability, splicing, transport, and translation of mRNAs; and how they function in the response of plants to developmental and environmental signals.

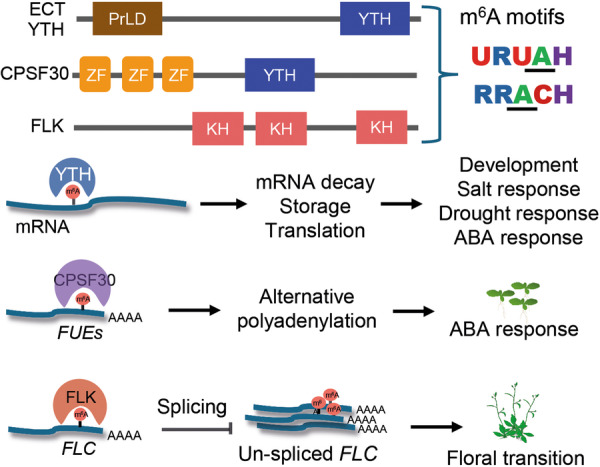

## INTRODUCTION

Chemical modifications in RNAs, including mRNA, rRNA, tRNA, microRNA, and long noncoding RNA, have recently been recognized as a potent gene regulatory mechanism in living organisms (Boccaletto et al., 2022; reviewed by Hu et al., 2022; Tang et al., 2023). More than 170 chemical modifications have been identified in RNAs, with *N* ^6^‐methyladenosine (m^6^A) being the most abundant modification in eukaryotic mRNAs (Boccaletto et al., 2022). The m^6^A mark is added, removed, and interpreted by methyltransferases (referred to as “writers”), demethylases (referred to as “erasers”), and RNA‐binding proteins (referred to as “readers”), respectively. In plants, the m^6^A writer components have been identified and characterized by analyzing the mutant of corresponding genes; for instance, methyltransferase A (MTA) and MTB function as catalytic m^6^A writers, and VIRILIZER (VIR), FKBP12‐interacting protein 37 (FIP37), ubiquitin E3 ligase (HAKAI), and HAKAI‐interacting zinc finger protein 2 (HIZ2) function as auxiliary components in Arabidopsis (*Arabidopsis thaliana*) (Zhong et al., 2008; Bodi et al., 2012; Růžička et al., 2017; Zhang et al., 2022). In addition, an independent methyltransferase FIONA1 (FIO1) has been identified in Arabidopsis (Wang et al., 2022; Xu et al., 2022; Cai et al., 2023). The alpha‐ketoglutarate‐dependent dioxygenase homolog (ALKBH) proteins have been identified and characterized as m^6^A erasers by analyzing the loss‐of‐function mutant of corresponding genes in plants: for instance, ALKBH9B, ALKBH9C, and ALKBH10B in Arabidopsis (Duan et al., 2017; Martínez‐Pérez et al., 2017; Amara et al., 2022; Tang et al., 2021, 2022), ALKBH2 in tomato (*Solanum lycopersicum*) (Zhou et al., 2019a), ALKBH9 in rice (*Oryza sativa*) (Tang et al., 2024), and ALKBH10 in cotton (*Gossypium hirsutum*) (Cui et al., 2022). The reversible activity of these m^6^A writers and erasers determines the overall levels of m^6^A within the conserved sequences of the RRACH, URUAH, and UACAGAG motifs (R and H denote A/G and A/C/U, respectively, while the underlined A is the m^6^A site) (Duan et al., 2017; Zhou et al., 2019a; Hu et al., 2021; Xu et al., 2022; Cai et al., 2023; Tang et al., 2024). Although m^6^A readers have attracted less attention than m^6^A writers and erasers, several recent studies have highlighted the significance of m^6^A readers in gene regulation during plant development and response to environmental cues (Table 1), which expands our understanding of the cellular role of m^6^A readers in plants. This review discusses the latest development in m^6^A reader research, emphasizing the diverse RNA‐binding domains crucial for m^6^A reader function and the biological and cellular role of m^6^A readers in plant development and stress responses. Moreover, we propose potential future research directions and challenges to identify novel m^6^A readers and elucidate the cellular and mechanistic role of m^6^A readers in plants.

**Table 1:** The identified mRNA m^6^A readers in plants.

Plant species	Name	Function	Cellular role	Reference
Arabidopsis (*A. thaliana*)	ECT1	SA response and defense	Phase separation	Lee et al. (2024)
	ECT2/3/4	Trichome development	mRNA stability	Scutenaire et al. (2018)
		Morphogenesis		Arribas‐Hernández et al. (2018)
		ABA response		Wei et al. (2018)
				Song et al. (2023)
	ECT8	ABA response	Phase separation	Wu et al. (2024)
		Salt response	mRNA decay	Cai et al. (2024b)
	ECT9	Pathogen defense	Phase separation	Wang et al. (2023a)
	ECT12	Salt and drought response	mRNA stability	Amara et al. (2024)
	CPSF30‐L	Nitrate signaling	Polyadenylation	Hou et al. (2021)
		Flowering and ABA response	Phase separation	Song et al. (2021)
	FLK	Floral transition	mRNA stability splicing	Amara et al. (2023)
Rice (*Oryza sativa*)	YTH07	Flowering	Repress protein accumulation	Cui et al. (2024)
Tomato (*Solanum lycopersicum*)	YTH2	Fruit aroma production	Phase separation	Bian et al. (2024)
	YTP8/9	Low‐temperature, waterlogging	(Not determined)	Zhang et al. (2024)
Apple (*Malus domestica*)	YTP2	Powdery mildew resistance	Translation efficiency	Guo et al. (2022)

### RNA‐binding domains exhibiting potential m^6^A reader function

Among the potential m^6^A readers, the YT521‐B homology (YTH) domain proteins are the most prevalent and well characterized m^6^A readers in plants and animals. The YTH domain, first identified in the splicing factor YT521‐B, is characterized by conserved residues within an α‐helix/β‐sheet structure and serves as a novel RNA‐binding domain that identifies m^6^A marks in a methylation‐dependent manner (Zhang et al., 2010). For occurrence, domain structures, and binding mode of YTH proteins in animals, refer to the review paper by Patil et al. (2018). The YTH domain possesses a hydrophobic methyl‐binding pocket that significantly increases its affinity for m^6^A‐modified RNA (Li et al., 2014; Theler et al., 2014; Xu et al., 2014; Zhu et al., 2014). Upon interaction between the YTH domain and RNA, the YTH protein undergoes a conformational shift from a disordered to an ordered state, increasing the stability of the YTH domain and augmenting its RNA‐binding affinity (Stowell et al., 2018). The YTH domain protein in Arabidopsis was first identified as a novel protein that is specifically associated with the calcineurin B‐like‐interacting protein kinase 1 (CIPK1) (Ok et al., 2005). Based on the presence of a highly similar C‐terminal region, these proteins are named “the evolutionarily conserved C‐terminal (ECT) region” (Ok et al., 2005; Li et al., 2014; Scutenaire et al., 2018).

**Figure 1:**
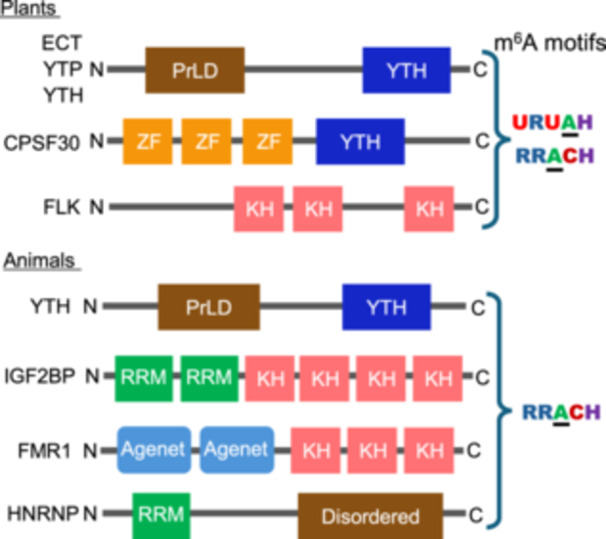
The domain structures of m^6^A reader proteins and the m^6^A motifs recognized by m^6^A readers Schematic domain structures of ECT, YTP, CPSF30, and FLK in plants and YTH, IGF2BP, FMR1, and HNRNP in animals: YTH, YT521‐B homology; KH, K‐homology; RRM, RNA‐recognition motif; ZF, zinc finger; PrLD, prion‐like domain. The RRACH motifs are found in both plant and animal mRNAs, whereas the URUAH motifs are found only in plant mRNAs. R and H represent G/A and A/C/U, respectively. The underlined As represents the m^6^A modification sites.

Previous animal studies demonstrated that the YTH proteins bind to RRm^6^ACH motifs (Wang et al., 2016; Wu et al., 2017; Liao et al., 2018). In a further plant study, ECT2 was found to bind to the UGUm^6^AY motif, a plant‐specific m^6^A consensus motif (Wei et al., 2018) (Figure 1). In addition, ECT2 also displayed a binding preference for RRm^6^ACH motifs (Arribas‐Hernández et al., 2021a). In addition, oligo‐(U) and UNUNU motifs upstream of m^6^A sites contribute to ECT2 binding via its intrinsically disordered region (IDR). Furthermore, URUAH motifs were observed to act as potential sites of competition between ECT2 and other regulatory proteins (Arribas‐Hernández et al., 2021a). Moreover, significant overlapping between ECT2 and ECT3 targets suggests functional redundancy (Arribas‐Hernández et al., 2021b). A recent study indicated that the YTH domains in all Arabidopsis ECT proteins possessed m^6^A‐binding capability and that lineage‐specific neofunctionalization of ECT1, ECT9, and ECT11 occurred following late duplication events involving altered properties of both the YTH domains and IDRs (Flores‐Téllez et al., 2023). Considering that the genomes of rice (*Oryza sativa*), tomato (*Solanum lycopersicum*), and wheat (*Triticum aestivum*) harbor 12, 9, and 39 YTH domain genes, respectively (Yin et al., 2021), it will be interesting to explore the similarities and differences in domain structure, binding activity, and sequence preference of each YTH protein in different plant species.

In addition to the YTH proteins, diverse RNA‐binding proteins (RBPs) harboring different RNA‐binding domains have been demonstrated to function as m^6^A readers. For instance, eIF3A, a subunit of the translation initiation complex eIF3 that contains an RNA‐recognition motif (RRM), acts as an m^6^A reader in animals (Meyer et al., 2015; Valášek et al., 2017). Recent mass spectrometry‐based screening of the m^6^A‐interacting RBPs in mammalian cells has allowed the identification of various potential m^6^A readers, including K‐homology (KH) proteins, CCHC zinc finger proteins, RRM proteins, and zinc knuckle proteins, as well as the YTH domain proteins (Edupuganti et al., 2017; Huang et al., 2018). Fragile X mental‐retardation protein (FMR1), which contains two Agenet domains, three KH domains, and one RGG domain (Figure 1), has been demonstrated as an m^6^A reader (Edupuganti et al., 2017). Furthermore, insulin‐like growth factor 2 binding protein (IGF2BP1), which harbors two RRMs and four KH domains (Figure 1), has been determined to recognize the consensus GGm^6^AC motifs (Huang et al., 2018). Moreover, heterogeneous nuclear ribonucleoprotein A2B1 (HNRNPA2B1), HNRNPC, and HNRNPG, which contains an RRM (Figure 1), were characterized as potential m^6^A readers in animals (Alarcón et al., 2015; Geuens et al., 2016). Although these RBPs as m^6^A readers have not been identified in plants, bioinformatic analyses of homolog search revealed that the Arabidopsis and rice genomes encode potential orthologs of these animal proteins (Table S1). A recent study revealed that FLOWERING LOCUS K (FLK), an Arabidopsis ortholog of human IGF2BP (Figure 1), is a novel mRNA m^6^A reader in plants (Amara et al., 2023). It will be of keen interest to determine whether these RBPs harboring particular domains function as m^6^A readers in plants.

### The biological function of m^6^A readers in plants

#### Development and morphogenesis

**Figure 2:**
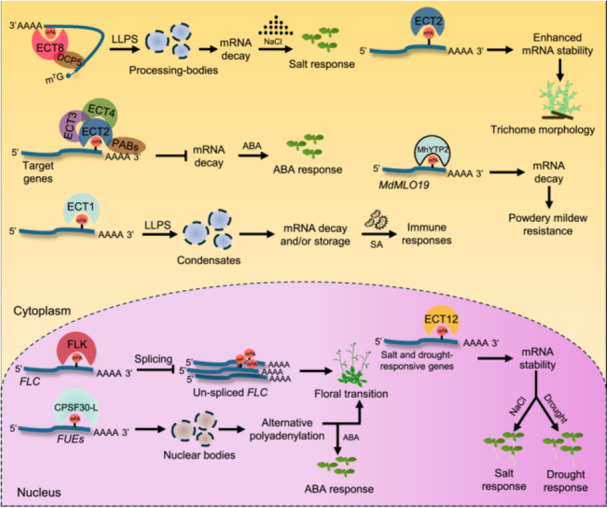
The molecular and cellular roles of m^6^A readers regulating mRNA stability, alternative polyadenylation, and alternative splicing In the cytoplasm, ECT8 regulates salt response through DECAPPING 5 (DCP5)‐mediated mRNA decay in processing bodies. The ECT2/ECT3/ECT4 complex stabilizes target genes by interacting with poly(A)‐binding proteins (PABs), leading to an ABA response. ECT1 regulates the salicylic acid (SA) and pathogen response through liquid–liquid phase separation (LLPS) to control mRNA decay and storage. ECT2 enhances the stability of m^6^A‐modified mRNAs, thereby controlling trichome morphology. The apple MhYTP2 promotes the degradation of *MdMLO19* mRNAs and enhances powdery mildew resistance. In the nucleus, CPSF30‐L recognizes m^6^A‐modified far‐upstream elements (FUEs) to control alternative polyadenylation by forming nuclear bodies, which modulate floral transition and ABA response. FLK binds to the m^6^A‐modified *FLC* to reduce splicing, leading to early flowering. ECT12 plays a role in the salt and drought stress response by regulating the stability of the m^6^A‐modified salt‐ and drought‐responsive genes.

**Figure 3:**
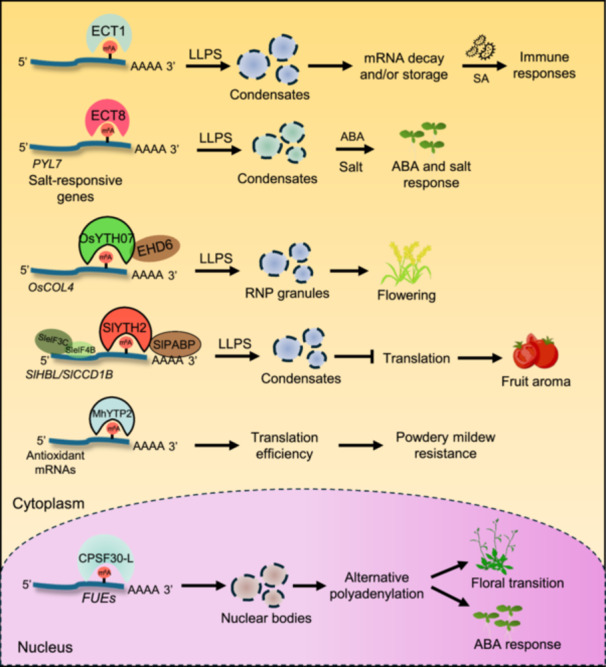
The molecular and cellular roles of m^6^A readers regulating translation and phase separation In the cytoplasm, ECT1 undergoes liquid–liquid phase separation (LLPS) to regulate mRNA decay and storage, leading to salicylic acid (SA) and pathogen response. ECT8 regulates salt and ABA response through LLPS to sequester *PYL7*, an ABA receptor, and salt‐responsive genes. The rice YTH07‒early heading date 6 (EHD6) complex binds to and sequesters *OsCOL4* mRNA to ribonucleotide protein (RNP) granules through LLPS to promote flowering. The tomato SlYTH2 binds to *SlHPL* and *SlCCD1B* transcripts and forms cytosolic condensates with translational regulators SlelF3C and SlelF4B to inhibit aroma production. The apple MhYTP2 promotes the translation of antioxidant genes and enhances powdery mildew resistance. In the nucleus, CPSF30‐L enhances the formation of liquid‐like nuclear bodies to control alternative polyadenylation, which modulates floral transition and ABA response.

The alteration or interpretation of m^6^A marks in mRNAs is associated with significant phenotypic variations, including embryonic lethality and organ‐specific developmental defects (Figures 2, 3). The molecular link between m^6^A and plant development was first discovered from the analysis of loss‐of‐function *ect* mutants in Arabidopsis: the m^6^A binding function of ECT2 is required for normal trichome morphology (Wei et al., 2018). Subsequently, the role of ECT2 in leaf and silique development was further confirmed by the analysis of ECT2‐overexpressing transgenic plants (Wu et al., 2020). Additional studies have demonstrated that ECT2, ECT3, and ECT4 are expressed at leaf formation sites in the shoot apex of young seedlings and in the division zone of developing leaves, which is implicated in modulating plant organogenesis (Arribas‐Hernández et al., 2018; Tankmar et al., 2023). Furthermore, ECT2/ECT3/ECT4 are primary mediators of m^6^A‐stimulated proliferation of primed stem cells in organ primordia (Arribas‐Hernández et al., 2020). The cleavage and polyadenylation specificity factor 30 (CPSF30), one of the 13 Arabidopsis YTH family proteins, was determined as an m^6^A reader whose m^6^A‐binding activity is required for the floral transition by controlling the choice of the polyadenylation site on the transcripts involved in the floral transition (Song et al., 2021). In contrast, ECT12 was shown to have no impact on normal Arabidopsis growth and development (Amara et al., 2024).

The role of m^6^A readers in the development of crops also continues to emerge. A recent study in rice has demonstrated that the YTH07 and early heading date 6 (EDH6) facilitate efficient m^6^A binding and repress OsCOL4 protein accumulation, which is crucial for flowering time control (Cui et al., 2024). In addition, a recent study in tomatoes has revealed that the loss‐of‐function *SlYTH1* mutant exhibited pleiotropic phenotypes, including delayed seed germination, shortened seedling root, retarded plant growth and development, and elongated and flattened fruits with reduced locule number (Yin et al., 2022). Moreover, SlYTH2 in tomatoes was determined as an m^6^A reader, and the CRISPR‒Cas9‐mediated loss‐of‐function *slyth2* mutants exhibited an increased production of aroma‐associated volatiles, suggesting that SlYTH2 negatively regulates tomato fruit aroma (Bian et al., 2024).

Apart from the YTH domain proteins, the role of other potential m^6^A readers in plants remains largely unexplored. However, a recent study demonstrated that FLK, an RBP that harbors three KH domains, is a novel mRNA m^6^A reader that regulates Arabidopsis flowering (Amara et al., 2023). Collectively, these findings indicate that m^6^A readers, including YTH and KH domain proteins, are crucial for plant organogenesis, morphogenesis, and controlling flowering time.

#### Abiotic stress response

Epitranscriptomic regulation is a potent strategy to fine tune gene regulation rapidly and efficiently in plants challenged by adverse and harsh environmental stresses. Numerous recent studies have elucidated the involvement of m^6^A writers and erasers in plant responses to diverse stresses (reviewed by Hu et al., 2022; Zhou et al., 2022; Tang et al., 2023). Notably, the crucial role of m^6^A readers in the Arabidopsis response to abiotic stress, abscisic acid (ABA), and nutrients has recently started to emerge (Figures 2, 3). The m^6^A recognition sites in the YTH domain of CPSF30‐L are crucial for regulating nitrate content, assimilation, and signaling (Hou et al., 2021). In addition, ECT12 impacts the seedling growth and survival of Arabidopsis in response to salt or drought stress (Amara et al., 2024). Recent studies have demonstrated that *ect8* mutants are tolerant to drought stress but sensitive to salt stress, indicating that ECT8 plays a role as a negative and positive regulator in drought and salt stress, respectively (Cai et al., 2024a; Wu et al., 2024). Moreover, the *cpsf30‐l* and *ect8* mutants displayed a retarded seed germination and less cotyledon greening in response to ABA than the wild type, indicating that CPSF30‐L and ECT8 undertake positive regulatory roles during ABA response (Song et al., 2021; Wu et al., 2024). Furthermore, by analyzing the *ect2/4* and *ect3/4* double mutants as well as the *ect2/3/4* triple mutants, it was demonstrated that ECT2, ECT3, and ECT4 interact with each other and perform genetically redundant functions in ABA response during seed germination and post‐germination growth (Song et al., 2023).

The role of m^6^A readers in the response of crops to abiotic stress is currently emerging. Genome‐wide identification of YTH domain proteins in navel orange (*Citrus sinensis*) revealed 10 CitYTH genes that displayed distinct expression patterns under heat, cold, salt, and drought stresses (Ouyang et al., 2019), suggesting the differential role of CitYTHs in the response of navel orange to various stresses. In addition, nine YTH domain‐containing RNA‐binding proteins (YTPs) were identified in tomatoes, among which the overexpression of *SlYTP8* increased the sensitivity of tomato plants to low‐temperature stress (Zhang et al., 2024). In contrast, the overexpression of *SlYTP9* increased the resistance of tomatoes to stress induced by waterlogging (Zhang et al., 2024). These findings collectively indicated that m^6^A readers are crucial for the response of plants to abiotic stresses.

#### Biotic stress response

Many recent studies have elucidated the significant role of m^6^A writer‐mediated or eraser‐mediated m^6^A modification in the response of plants to biotic stresses (reviewed by Yue et al., 2022; Zhou et al., 2022; Tang et al., 2023). Although our understanding of the role that m^6^A readers perform in the biotic stress response is lower than the abiotic stress response, recent research has shed light on the significance of m^6^A readers in the plant response to biotic stress (Figures 2, 3). In Arabidopsis, the *ect1/9* double mutants showed higher resistance to the virulent bacterial pathogen *Psm* ES4326, suggesting that ECT9 and ECT1 negatively affect plant immunity (Wang et al., 2023a). From the analysis of the single, double, and triple *ect2*, *ect3*, *ect4*, and *ect5* mutants, it was demonstrated that ECT2, ECT3, and ECT5 m^6^A‐binding activity is required for antiviral activity (Martínez‐Pérez et al., 2023). Notably, the *ect1* and *ect2* mutants are sensitive to salicylic acid (SA) and the growth of *Pseudomonas syringae* pv. *tomato* (*Pst*) DC3000 decreased in the *ect1* mutant but increased in ECT1‐overexpressing plants, indicating that ECT1 negatively regulates the plant defense response against *Pst* DC3000 (Lee et al., 2024). The powdery mildew resistance was also enhanced by the overexpression of MhYTP2, an ortholog of Arabidopsis ECT2 in apples (*Malus* × *domestica* Borkh.) (Guo et al., 2022). These findings collectively indicated that m^6^A readers are crucial cellular components associated with the immune response in plants.

### Cellular role of m^6^A readers in plants

#### The stabilization and decay of mRNAs

The stability of m^6^A‐containing mRNAs is governed by the intricate interplay between m^6^A modification and various cellular factors, including RBPs, RNA structures, and other modification types. Early studies in mammals revealed a correlation between the increased mRNA half‐life and the inhibition of m^6^A modification or downregulation of the m^6^A writer complex (Batista et al., 2014; Wang et al., 2014). A recent study has demonstrated that HNRNPC enhances the stability of mRNA through the MAPK pathway (Chen et al., 2024). Several animal studies have uncovered that the decay of m^6^A‐containing mRNAs depends on m^6^A readers through two distinct pathways: YTHDF2‒CCR4‒NOT and YTHDF3‒PAN2‒PAN3 (Du et al., 2016; Kretschmer et al., 2018; Liu et al., 2020).

In plants, combined m^6^A‐seq and RNA‐seq analyses of m^6^A writer or eraser mutants have been conducted to elucidate the overall impact of m^6^A on mRNA stability (Duan et al., 2017; Hu et al., 2021; Cai et al., 2024b). Importantly, the stability of mRNAs is determined by specific m^6^A readers (Figure 2). For instance, ECT2 enhances the stability of the target m^6^A‐modified mRNAs (Wei et al., 2018). Moreover, ECT2 interacts directly with poly(A) binding proteins PAB2 and PAB4, thereby contributing to the increased stability of m^6^A‐modified mRNAs (Song et al., 2023). FLK binds to the m^6^A site located in the 3ʹ‐untranslated region (UTR) of *FLC* transcripts and accelerates the decay of *FLC* mRNA, leading to early flowering (Amara et al., 2023). ECT12 upregulates and downregulates the stability of the mRNAs of positive and negative effectors, respectively, involved in salt or drought stress, conferring salt or drought tolerance (Amara et al., 2024). CPSF30‐L controls polyadenylation site choice, which stabilizes the mRNAs involved in the ABA response (Song et al., 2021).

The remaining key question is how m^6^A readers control the stability and decay of the target transcripts. Notably, a recent study demonstrated that ECT8 physically interacts with the decapping protein DECAPPING 5 (DCP5), and the ECT8‒DCP5 complex facilitates the degradation of the m^6^A‐modified mRNAs of salt stress negative regulators, leading to salt tolerance (Cai et al., 2024b). Considering the m^6^A reader‐mediated deadenylation and endoribonucleolytic cleavage of mRNAs observed in animals (Du et al., 2016; Kretschmer et al., 2018; Liu et al., 2020), the m^6^A reader‐mediated decapping of mRNAs represents a novel mechanism that underlies the regulatory role of m^6^A modification in mRNA stabilization and decay. It will be interesting to determine further whether other m^6^A readers interact with specific proteins associated with decapping, deadenylation, and endoribonucleolytic cleavage, leading to mRNA cleavage in different plant species. In addition, determining whether m^6^A readers recruit particular proteins that protect the m^6^A reader‐bound mRNAs from cleavage will greatly increase our understanding of the mechanistic role of m^6^A readers in mRNA stabilization.

#### Alternative polyadenylation

Alternative polyadenylation (APA) is a potent mechanism of gene regulation, and the m^6^A role in regulating APA is soon emerging. A previous study in animals has demonstrated that VIRMA, an m^6^A writer, associates with polyadenylation cleavage factors CPSF5 and CPSF6 to regulate 3′UTR length and APA (Yue et al., 2018). A nuclear m^6^A reader YTHDC1 in animals regulates APA, altering 3′UTR length (Kasowitz et al., 2018). A further study revealed that YTHDC1 suppresses proximal APA sites and produces longer 3′UTR transcripts by binding to their upstream m^6^A sites, supporting the direct role m^6^A readers perform in regulating APA (Chen et al., 2022).

Currently, the link between m^6^A modification and APA in plants has only been explored in Arabidopsis (Figures 2, 3). A previous study has demonstrated that VIR‐mediated m^6^A methylation regulates the mRNA stability of several salt stress effectors by affecting 3ʹUTR lengthening linked to APA (Hu et al., 2021). The direct role of plant m^6^A readers in APA was first determined in a study that showed that CPSF30‐L mediates a poly(A) site selection shift in numerous nitrate signaling‐related transcripts (Hou et al., 2021). An additional study demonstrated that CPSF30‐L regulates APA by binding to the UGUARNN and GAAMH motifs, located 35‒130 nt upstream of the poly(A) site within the far‐upstream elements (FUE), and to the SAAUAAA motifs, situated 10–35 nt upstream within the near‐upstream element (NUE) region (Song et al., 2021) (Figure 2). Collectively, these findings indicated that m^6^A readers are crucial for APA and gene regulation in plants.

#### Alternative splicing

Alternative splicing (AS) is an efficient process that generates multiple protein products from a single transcript, thereby expanding the information repertory of a gene, which plays a crucial role in plant development and stress response (reviewed by Liu et al., 2024). The crucial role of m^6^A in regulating AS is emerging. Previous animal studies have demonstrated that YTHDC1 regulates AS through recruiting pre‐mRNA splicing factors SRSF3, SRSF7, and CPSF6, providing direct evidence for the role of m^6^A readers in regulating mRNA splicing (Xiao et al., 2016; Kasowitz et al., 2018). In addition, HNRNPG uses RGG to co‐transcriptionally interact with both RNA polymerase II and m^6^A‐modified nascent pre‐mRNA, thereby modulating RNA polymerase II occupancy and AS (Zhou et al., 2019b).

The role of m^6^A readers in splicing precursor‐mRNAs (pre‐mRNAs) in plants remains largely unexplored. A recent study has demonstrated that FIO1, an m^6^A writer in Arabidopsis, regulates the splicing of many pre‐mRNAs through U5 and U6 snRNA‐mediated splice site selection (Parker et al., 2022). In addition, FIO1‐mediated m^6^A modification in flowering locus C (*FLC*), a key floral repressor, is crucial for splicing of *FLC* to regulate floral transition (Cai et al., 2023). These findings indicated that m^6^A modification is tightly associated with pre‐mRNA splicing. Notably, a recent study highlighted the crucial link between an m^6^A reader and splicing in plants; FLK, a novel mRNA m^6^A reader in Arabidopsis, binds to m^6^A‐modified *FLC* mRNA and is indispensable for the accurate splicing of *FLC* transcripts (Amara et al., 2023) (Figure 2). However, whether m^6^A modification is associated with AS in plants and how m^6^A readers regulate AS in plant development and response to environmental cues remains unexplored.

#### Translation control

m^6^A modification in mRNAs either positively or negatively influences translation depending on the location of m^6^A marks, such as 5′UTR, 3′UTR, and coding regions, in animals (reviewed by Rodriguez et al., 2022). Several animal studies have demonstrated that m^6^A readers play a crucial role in translation. For instance, human YTHDF1 actively facilitates protein synthesis using translation machinery to enhance translation efficiency (Wang et al., 2015). In addition, YTHDF3 and YTHDF1 promote the translation of their target mRNAs (Li et al., 2017; Shi et al., 2017). Moreover, YTHDC2 plays a pivotal role in spermatogenesis by enhancing the translation efficiency of its target genes (Hsu et al., 2017). Considering that the export of mRNA from the nucleus to the cytoplasm affects translation of the mRNA, it is interesting to note that YTHDC1 mediates the export of m^6^A‐modified mRNAs from the nucleus to the cytoplasm in HeLa cells (Roundtree et al., 2017).

The role of m^6^A in plant translation control is just beginning to be discovered. A previous study has demonstrated, through a parallel analysis of the transcriptome‐wide mRNA m^6^A distribution and polysome profiling, that transcripts with m^6^A marks around the start codon show a high translational status, raising an intriguing possibility that the m^6^A marks near the start codon are associated with enhanced mRNA translation (Luo et al., 2020). In addition, a recent study employed a combined analysis of the m^6^A methylome, transcriptome, and translatome of the wild type and *mta* mutant and revealed that the m^6^A‐containing mRNAs generally have higher translation efficiency than non‐m^6^A‐containing mRNAs under normal and low temperatures (Wang et al., 2023b). Another recent study employing ribosome profiling (ribo‐seq) demonstrated no significant differences in translation efficiency between ECT2 or ECT2‐m^6^A targets and non‐targets in wild type, *ect2*, and *ect2/3/4* mutants, suggesting that ECT2/ECT3/ECT4 have no function in protein translation (Song et al., 2023).

The m^6^A reader MhYTP2 in apples regulates the translation efficiency of antioxidant genes, conferring powdery mildew resistance (Guo et al., 2022) (Figure 3). Notably, the role of m^6^A readers in translation control in plants has been recently demonstrated: SlYTH2, a newly identified m^6^A reader in tomatoes, was shown to sequester m^6^A‐modified transcripts, including *SlHPL* and *SlCCD1B*, into liquid‐like condensates, potentially impeding polysome assembly and translation of target mRNAs (Bian et al., 2024) (Figure 3). Hence, it will be interesting to determine whether other m^6^A readers regulate translation efficiency in plants and to explore whether m^6^A reader‐mediated translation control varies depending on the position of m^6^A marks in a transcript.

#### Liquid‒liquid phase separation

Liquid‒liquid phase separation (LLPS) is a pivotal process governing plant development and stress responses (reviewed by Field et al., 2023; Solis‐Miranda et al., 2023). Many animal studies have demonstrated that YTHD m^6^A readers play a crucial role in phase separation (Ries et al., 2019; Fu and Zhuang, 2020; reviewed by Kang and Xu, 2023). Moreover, it was demonstrated that the N‐terminal regions of YTHDF proteins contain potential prion‐like domains (PrLDs) and IDRs, which facilitate the formation of various biomolecular condensates, including processing bodies (PBs), stress granules (SGs), and ribonucleoprotein (RNP) granules, through LLPS (reviewed by Sikorski et al., 2023).

The significance of m^6^A readers in phase separation in plants is just beginning to be unraveled. The m^6^A‐binding activity of CPSF30‐L enhances the formation of liquid‐like nuclear bodies, which primarily recognize the m^6^A‐modified FUEs to regulate polyadenylation site selection (Song et al., 2021) (Figure 3). ECT2‒8 and ECT10 in Arabidopsis were shown to contain putative PrLDs, and the binding of ECT2/3/4 to the m^6^A‐modified transcripts could drive LLPS in plant cells (Lee et al., 2022). Moreover, genetic complementation of the different domains of ECT family members to the *ect2/3/4* mutant demonstrated that the divergent function of ECT members is caused mainly by the properties of IDRs (Flores‐Téllez et al., 2023). ECT9 exhibits LLPS, and its ability to form condensates is diminished following SA treatment (Wang et al., 2023a). Although ECT1 cannot independently form condensates, it can be recruited to ECT9 condensates, which negatively affects plant immunity (Wang et al., 2023a). Notably, ECT1 forms PBs and SGs in response to SA treatment, which aids in the degradation of m^6^A‐modified mRNAs associated with the SA response (Lee et al., 2024). In a recent study, ECT8 was shown to act as a driver for LLPS to sequester the m^6^A‐modified ABA receptor gene *PYRABACTIN RESISTANCE 1‐LIKE 7* (*PYL7*) into SGs, which provides a negative feedback regulation of ABA signaling (Wu et al., 2024) (Figure 3).

Apart from Arabidopsis, the role of m^6^A readers in LLPS was recently determined in rice and tomato plants. YTH07, a novel m^6^A reader in rice, interacts with early heading date 6 (EHD6), a flowering‐promoting gene, and the YTH07‒EHD6 complex binds to the *CONSTANS‐like 4* (*OsCOL4*) mRNA, a flowering repressor, and undergoes LLPS to sequester *OsCOL4* into RNP granules, leading to early flowering (Cui et al., 2024) (Figure 3). Additionally, SlYTH2, an m^6^A reader in tomato plants, undergoes LLPS and sequesters target m^6^A‐RNAs, including *SlHPL* and *SlCCD1B* that are associated with volatile synthesis, into condensates to regulate the synthesis of fruit aroma (Bian et al., 2024) (Figure 3). These discoveries highlight the crucial role of m^6^A reader‐mediated LLPS in plant development, flowering, aroma production, and the SA and ABA responses.

### Techniques for identifying and characterizing m^6^A readers

**Figure 4:**
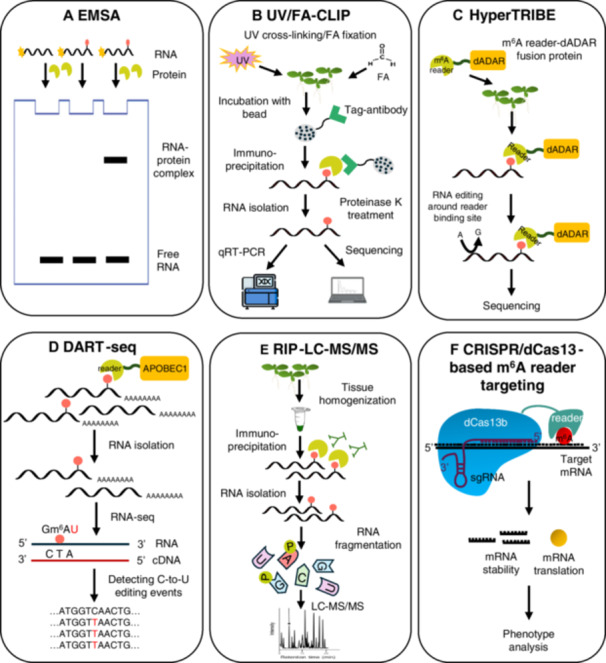
Techniques commonly used for identifying and characterizing m^6^A readers **(A)** Electrophoretic mobility shift assay (EMSA): The purified recombinant protein and the synthetic target RNA are mixed together, and the RNA‒protein complexes are separated on a non‐denaturing polyacrylamide gel and detected by an image analyzer. **(B)** UV/FA‐CLIP: The RNA‒protein complexes are fixed using UV or formaldehyde (FA) cross‐linking and immunoprecipitated using the antibody specific to the m^6^A reader protein. The RNAs bound to the m^6^A reader are recovered and subsequently analyzed by qRT‐PCR or sequencing. **(C)** HyperTRIBE: The m^6^A reader is fused to the *Drosophila* adenosine deaminase acting on the RNA (dADAR) domain, and this fusion protein is expressed in target plants, allowing ADAR to edit adenosine to guanosine in RNAs bound by the m^6^A reader. These edits are subsequently detected by RNA sequencing. **(D)** DART‐seq: The cytidine deaminase APOBEC1 is fused with the m^6^A reader to direct C‐to‐U editing at cytidine residues adjacent to m^6^A sites. APOBEC1‐m^6^A reader fusion protein is expressed, after which total RNA is extracted and analyzed using RNA sequencing. The presence of C‐to‐U mutations is used to pinpoint the locations of m^6^A mark at m^6^A reader binding site. **(E)** RIP‐LC–MS/MS: The tissues are homogenized, and the RNA‐m^6^A reader complex is immunoprecipitated using m^6^A reader‐specific antibody. The bound RNA is recovered, digested into nucleotide fragments, and analyzed using LC–MS/MS to identify and quantify RNA‐m^6^A reader interactions. **(F)** CRISPR/dCas13‐based m^6^A reader targeting: The m^6^A reader protein is fused with a catalytically inactive dCas13b protein, which can target the m^6^A reader to a specific m^6^A site within an mRNA using guide RNA complementarity. The binding of m^6^A reader to specific m^6^A mark can alter the stability or translation of the mRNA, leading to phenotype changes of the transgenic plants.

The development of efficient tools and techniques for detecting the levels and patterns of m^6^A modification and for identifying the m^6^A reader targets is a prerequisite for studying the m^6^A function. Several high‐throughput m^6^A sequencing techniques, including antibody‐dependent and antibody‐independent m^6^A‐sequencing methods, have been utilized in plant research. For detailed information regarding m^6^A detection tools, refer to the review papers by Prall et al. (2023) and Tang et al. (2023). Here, we introduce and describe the commonly used techniques to identify and confirm target mRNAs of m^6^A readers and to characterize m^6^A reader‒target RNA binding: electrophoretic mobility shift assay (EMSA), ultraviolet light/formaldehyde‐assisted cross‐linking and immunoprecipitation (UV/FA‐CLIP), HyperTRIBE (targets of RNA‐binding proteins identified by editing), DART‐seq (deamination adjacent to RNA modification targets‐seq), RIP‐LC–MS/MS (RNA immunoprecipitation‐liquid chromatography–mass spectrometry), and CRISPR/dCas13‐based m^6^A reader targeting (Figure 4).

#### EMSA

This technique is used to confirm the direct binding of the m^6^A reader to the putative target RNA *in vitro*. The recombinant m^6^A reader protein is purified, and the target RNA containing specific sequences is synthesized. After incubating the m^6^A reader and target RNA, the RNA‒protein complexes are separated on a non‐denaturing polyacrylamide gel, and the RNA‒protein complex is detected using an image analyzer (Figure 4A). This technique has been used to confirm the binding of ECT2, ECT8, ECT12, CPSF30‐L, MhYTP2, and OsYTH07 to the m^6^A‐modified target RNAs (Wei et al., 2018; Song et al., 2021; Guo et al., 2022; Amara et al., 2024; Cai et al., 2024b; Cui et al., 2024). Although this technique is an efficient way to confirm the direct binding of the m^6^A reader to the putative target RNA, the shortcoming is that it cannot be applied to m^6^A readers whose putative target RNAs are unknown.

#### UV/FA‐CLIP

This technique is a powerful tool for discovering and identifying the RNA targets of specific m^6^A readers *in planta*. The RNA‒protein complexes are fixed using UV or formaldehyde and then immunoprecipitated using the antibody specific to the m^6^A reader protein. After recovering the RNAs bound to the m^6^A reader, the identity and levels of the bound RNAs are analyzed by qRT‐PCR or sequencing (Figure 4B). This technique has been widely used to discover and identify the target mRNAs of ECT1, ECT2, ECT8, CPSF30‐L, MhYTP2, and OsYTH07 (Song et al., 2021; Guo et al., 2022; Tankmar et al., 2023; Cai et al., 2024b; Cui et al., 2024; Lee et al., 2024; Wu et al., 2024).

#### HyperTRIBE

The proximity‐labeling method HyperTRIBE (targets of RNA‐binding proteins identified by editing) is a powerful genetic tool that identifies *in vivo* targets of RBPs (McMahon et al., 2016). This technique uses a fusion of a RBP of interest to the catalytic domain of the *Drosophila* adenosine deaminase acting on RNA (dADAR) to obtain an A to G mutation in mRNAs bound by the RBP of interest. These edits are subsequently detected by RNA sequencing (Figure 4C). This technique has been used to identify and reveal the overlapping mRNA targets of ECT2 and ECT3 in Arabidopsis (Arribas‐Hernández et al., 2021a, 2021b). Although this tool has not yet been widely applied to plant systems, the combined use of HyperTRIBE and UV/FA‐CLIP is highly valuable in confirming the binding of the m^6^A reader to the target mRNA *in planta*.

#### DART‐seq

This technique is an antibody‐free method for detecting m^6^A sites and can be used to confirm the binding of m^6^A readers to its targets in living cells (Meyer, 2019). The cytidine deaminase APOBEC1 is fused with the m^6^A reader to direct C‐to‐U editing at cytidine residues adjacent to m^6^A sites. APOBEC1‐m^6^A reader fusion protein is expressed in plant cells, and total RNA is extracted and analyzed using RNA sequencing. The presence of C‐to‐U mutations is used to pinpoint the locations of m^6^A marks at the m^6^A reader binding site (Figure 4D).

#### RIP‐LC–MS/MS

This technique is a precise tool for quantifying RNA–m^6^A reader interactions. The tissues are homogenized, and the RNA–m^6^A reader complex is immunoprecipitated using an m^6^A reader‐specific antibody. The bound RNA is recovered, digested into nucleotide fragments, and analyzed using LC–MS/MS to identify and quantify RNA–m^6^A reader interactions (Figure 4E).

#### CRISPR/dCas13‐based m^6^A reader targeting

This technique is a powerful tool to determine the function of the m^6^A reader in cells. The m^6^A reader protein is fused with a catalytically inactive dCas13b protein, which can target the m^6^A reader to a specific m^6^A site within an mRNA using guide RNA complementarity (Rauch et al., 2018). The binding of the m^6^A reader to a specific m^6^A mark can alter the stability or translation of the mRNA, leading to phenotype changes in the transgenic plants (Figure 4F).

### Outlook and future challenges

The identification and characterization of the m^6^A machinery in plants, including m^6^A writers and erasers, during the last several years have expanded our understanding of the transcriptome‐wide distribution of m^6^A methylome and the biological function of m^6^A modification in plant development and stress responses. Considering that the fate of m^6^A‐modified mRNAs depends on the degree and location of m^6^A marks in a transcript, it is imperative to determine how the m^6^A marks are interpreted by m^6^A readers to fully understand the biological and cellular role of m^6^A in regulating mRNA fate. With the recent advancement of the powerful technologies and novel tools described above, we are witnessing rapid progress in discovering novel m^6^A readers in diverse plant species, including Arabidopsis, rice, tomato, and apple. The m^6^A reader‐mediated interpretation of m^6^A marks is crucial for vegetative growth, flowering, fruit aroma production, and the response of plants to environmental cues, including abiotic and biotic stresses, SA, and ABA. However, many critical questions and challenges remain to further our understanding of the role of m^6^A modification in plants.

First, given that m^6^A methylation either increases or decreases mRNA stability (Duan et al., 2017; Hu et al., 2021; Cai et al., 2024b), it will be interesting to determine how the stability of transcripts is regulated by m^6^A reader binding and to investigate whether m^6^A readers recruit a particular protein that either protects or cleaves the m^6^A‐modified mRNAs.

Second, considering that the correct splicing of pre‐mRNAs requires specific sequences around the 5ʹ‐ and 3ʹ‐splice sites, as well as the structures of the pre‐mRNAs around these splice sites (reviewed by Lorković et al., 2000), it will be interesting to explore whether m^6^A readers regulate splicing by recognizing the m^6^A marks around the 5ʹ‐ and 3ʹ‐splice sites and to determine whether m^6^A readers recruit specific splicing factors necessary for the removal of introns.

Third, as the m^6^A marks near the start codon are associated with an enhanced mRNA translation (Luo et al., 2020), exploring whether m^6^A readers regulate the translation initiation or elongation of the transcribing m^6^A‐modified mRNAs through interacting with the translation initiation or elongation factors via an m^6^A position‐dependent manner will greatly deepen our understanding of the mechanistic role of m^6^A in translation control.

Fourth, given that the m^6^A reader‐mediated LLPS has emerged as a potent mechanism of gene regulation (reviewed by Kang and Xu, 2023), it will be of keen interest to discover whether binding of m^6^A readers to the target m^6^A‐modified mRNAs facilitates the phase separation of the m^6^A reader‒m^6^A mRNA complex to regulate the fate of the target mRNAs. In particular, exploring whether the m^6^A reader‐mediated LLPS varies depending on developmental and environmental cues will be crucial to understanding the cellular role of m^6^A readers in LLPS.

Fifth, considering that post‐translational modifications (PTMs), including phosphorylation, ubiquitination, and SUMOylation, are crucial for regulating protein localization, ligand or protein interactions, and SG assembly (Hofmann et al., 2021), it will be interesting to determine whether PTMs of m^6^A readers are associated with the functionality and phase separation potential of m^6^A readers in plants under different developmental and environmental cues.

Finally, as the understanding of the crosstalk between mRNA m^6^A modification and epigenetic regulators, including DNA methylation, histone modifications, microRNAs, long noncoding RNAs, and chromatin remodeling, has emerged in recent years (reviewed by Hu et al., 2024), it will be challenging to explore whether m^6^A readers play a role as a mediator to recruit particular protein factors associated with epigenetic regulators.

In summary, rapid progress in constructing and characterizing m^6^A writer and eraser mutants has unveiled the transcriptome‐wide distribution of m^6^A marks in mRNAs. Moreover, accumulating evidence has highlighted the crucial role of m^6^A reader‐mediated interpretation of m^6^A marks in epigenetic regulation of plant development and stress responses. Notwithstanding, many challenges remain in identifying and characterizing novel m^6^A readers in different plant species. In particular, it will be of keen interest to determine whether the other potential m^6^A readers, including KH proteins, CCHC zinc finger proteins, RRM proteins, and zinc knuckle proteins, identified by mass spectrometry‐based screening of m^6^A‐interacting RBPs in mammalian cells (Edupuganti et al., 2017; Huang et al., 2018), exist in plants, in addition to the well characterized YTH domain proteins. Moreover, deciphering the molecular and mechanistic role of m^6^A readers in regulating the stability, splicing, translation, and phase separation of target mRNAs in a developmental stage‐dependent or environmental signal‐dependent manner is worth exploring. With recent breakthroughs in CRISPR genome editing techniques, the m^6^A reader‐mediated interpretation of mRNA can be controlled using the CRISPR‒Cas13b‐fused reader approach. For instance, the dPspCas13b‒m^6^A reader system, in which the human m^6^A reader protein YTHDF1 or YTHDF2 is fused to a catalytically inactive PspCas13b protein, can target the reader to a specific m^6^A site within a particular mRNA (Rauch et al., 2018). Although this strategy is yet to be applied in plants, further development of this technique for plants will substantially boost the m^6^A reader‐mediated epitranscriptomics research. We anticipate exciting discoveries in the coming years.

## CONFLICTS OF INTEREST

The authors declare no conflict of interest.

## AUTHOR CONTRIBUTIONS

H.K. and T.K.H.N. perceived the ideas, analyzed the literature, and wrote the manuscript.

## Supporting information

Additional Supporting Information may be found online in the supporting information tab for this article: http://onlinelibrary.wiley.com/doi/10.1111/jipb.13781/suppinfo

**Table S1.** List of the potential mRNA m^6^A readers in plants
