# Supplementary material for: Reading m6A marks in mRNA: A potent mechanism of gene regulation in plants
Source: J Integr Plant Biol. 2024 Oct 4;66(12):2586–99. doi: 10.1111/jipb.13781 (PMC11622538; doi:10.1111/jipb.13781)
Supplement: Supplementary file 1 — Table S1. List of the potential mRNA m6A readers in plants [file JIPB-66-2586-s001.docx]

**Supplementary Table S1. List of the potential mRNA m^6^A readers in plants.**

| **m^6^A readers in animals** | **Domain** | **Reference** | **Potential plant ortholog^#^** | | |
| --- | --- | --- | --- | --- | --- |
|  |  |  | **Name** | **Gene ID** | **Function** |
| eIF3 | PCI/MPN domain | (Meyer et al., 2015; Yang et al., 2017) | AtEIF3A | At4G11420 | Pollen tube growth and embryogenesis, expansion of shoot apical meristem |
|  |  |  | OsEIF3A | Os01g0120800 | Organ size and pollen maturation |
| IGF2BP1/2/3 | RRM and KH domain | (Huang et al., 2018; Sun et al., 2019) | AtKH2 | At1G14170 | Cytokinin receptor |
|  |  |  | (KH domain containing protein) | Os09g0498600 | Unknown |
|  |  |  | (KH domain containing protein) | Os03g0627500 | Unknown |
| FMRP | KH and RGG domain | (Zhang et al., 2018; Edens et al., 2019) | AtGRP3 | At2G05520 | Root growth and aluminum response |
|  |  |  | AtALY1 | At5G59950 | mRNA transport, reproduction |
| Prrca2 | Arginine-glycine-rich domain | (Wu et al., 2019) | Not found | | |
| RBM45 | RRM domain | (Choi et al., 2022) | AtRBGD3 | At3G13224 | Unknown |
|  |  |  | (Similar to RNA binding protein 45) | Os03g0569900 | Unknown |
| HNRNPC | N-terminal RRM and C terminal-RBD domain (SRGP-rich low-complexity region) | (Liu et al., 2015) | AtRS41 | At5G52040 | Splicing factors, miRNA biogenesis |
|  |  |  | OsRS33 | Os02g0122800 | Splicing factors, abiotic stress responses |
| HNRNPG |  | (Liu et al., 2017) | AtGRP7 | At2G21660 | Abiotic stress responses, floral transition |
|  |  |  | AtGRP8 | At4G39260 | Root hair cell fate, phosphate starvation, flowering |
|  |  |  | OsGRP3 | Os03g0670700 | Drought response, thermos-tolerance |
| HNRNPA2B1 |  | (Wu et al., 2018) | (RNA-binding family protein) | At5G55550 | Unknown |
|  |  |  | LGG,  RBP-A | Os11g0637700 | Cell cycle and cell division in the rice spikelet hull |

^#^The potential m^6^A readers in plants were identified by homolog search using each animal protein as a query. At (*Arabidopsis thaliana*) and Os (*Oryza sativa*) genes are shown as a representative model plant for dicot and monocot, respectively.

**References**

**Choi, S.H., Flamand, M.N., Liu, B., Zhu, H., Hu, M., Wang, M.,** et al. (2022). RBM45 is an m^6^A-binding protein that affects neuronal differentiation and the splicing of a subset of mRNAs. Cell Rep. **40:** 111293.

**Edens, B.M., Vissers, C., Su, J., Arumugam, S., Xu, Z., Shi, H.,** et al. (2019). FMRP modulates neural differentiation through m^6^A-dependent mRNA nuclear export. Cell Rep. **28:** 845-854. e5.

**Huang, H., Weng, H., Sun, W., Qin, X., Shi, H., Wu, H.,** et al. (2018). Recognition of RNA N6-methyladenosine by IGF2BP proteins enhances mRNA stability and translation. Nat. Cell Biol. **20:** 285-295.

**Liu, N., Dai, Q., Zheng, G., He, C., Parisien, M., and Pan, T.** (2015). N6-methyladenosine-dependent RNA structural switches regulate RNA–protein interactions. Nature **518:** 560-564.

**Liu, N., Zhou, K.I., Parisien, M., Dai, Q., Diatchenko, L., and Pan, T.** (2017). N6-methyladenosine alters RNA structure to regulate binding of a low-complexity protein. Nucleic Acids Res. **45:** 6051-6063.

**Meyer, K.D., Patil, D.P., Zhou, J., Zinoviev, A., Skabkin, M.A., Elemento, O.,** et al. (2015). 5′ UTR m^6^A promotes cap-independent translation. Cell **163:** 999-1010.

**Sun, L., Fazal, F.M., Li, P., Broughton, J.P., Lee, B., Tang, L.,** et al. (2019). RNA structure maps across mammalian cellular compartments. Nat. Struct. Mol. Biol. **26:** 322-330.

**Wu, B., Su, S., Patil, D.P., Liu, H., Gan, J., Jaffrey, S.R.,** et al. (2018). Molecular basis for the specific and multivariant recognitions of RNA substrates by human hnRNP A2/B1. Nat. Commun. **9:** 1-12.

**Wu, R., Li, A., Sun, B., Sun, J.-G., Zhang, J., Zhang, T.,** et al. (2019). A novel m^6^A reader Prrc2a controls oligodendroglial specification and myelination. Cell Res. **29:** 23-41.

**Yang, Y., Fan, X., Mao, M., Song, X., Wu, P., Zhang, Y.,** et al. (2017). Extensive translation of circular RNAs driven by N6-methyladenosine. Cell Res. **27:** 626-641.

**Zhang, F., Kang, Y., Wang, M., Li, Y., Xu, T., Yang, W.,** et al. (2018). Fragile X mental retardation protein modulates the stability of its m^6^A-marked messenger RNA targets. Hum. Mol. Genet. **27:** 3936-3950.
